# Unraveling the effect of the combination of modified atmosphere packaging and ε-polylysine on the physicochemical properties and bacterial community of greater amberjack (*Seriola dumerili*)

**DOI:** 10.3389/fnut.2022.1035714

**Published:** 2022-11-18

**Authors:** Di Wang, Xupeng Li, Xianqing Yang, Shengjun Chen, Laihao Li, Yueqi Wang, Chuang Pan, Yongqiang Zhao

**Affiliations:** ^1^Key Laboratory of Aquatic Product Processing, Ministry of Agriculture and Rural Affairs of the People’s Republic of China, South China Sea Fisheries Research Institute, Chinese Academy of Fishery Sciences, Guangzhou, China; ^2^Co-Innovation Center of Jiangsu Marine Bio-Industry Technology, Jiangsu Ocean University, Lianyungang, China; ^3^Sanya Tropical Fisheries Research Institute, Sanya, China; ^4^Key Laboratory of Efficient Utilization and Processing of Marine Fishery Resources of Hainan Province, Sanya, China; ^5^Collaborative Innovation Center of Seafood Deep Processing, Dalian Polytechnic University, Dalian, China; ^6^Guangdong Agricultural Technology Extension Center, Department of Agriculture and Rural Affairs of Guangdong Province, Guangzhou, China

**Keywords:** greater amberjack, ε-polylysine, modified atmosphere packaging, shelf life extension, bacterial community

## Abstract

The combined effect of ε-polylysine (PL) and modified atmosphere packaging (MAP; 60% CO_2_/40% N_2_) on the bacterial community of greater amberjack filets and their physicochemical properties was evaluated at 4°C. The total viable counts (TVC), psychrotrophic bacterial count, sensory index, texture analysis, and total volatile basic nitrogen (TVB-N) revealed that PL, MAP, and MAP + PL treatment delayed the deterioration of greater amberjack filets. These treatment groups also showed decreased accumulation of biogenic amines. High-throughput 16S rRNA gene sequencing results indicated that these treatments suppressed the growth of *Pseudomonas* in greater amberjack filets. Furthermore, the MAP + PL treatment group was observed to be more effective than the PL and MAP groups, extending the shelf life of greater amberjack filets by 6 days. This investigation showed that the combination of PL and MAP has the potential to retain the quality and extend the shelf life of greater amberjack.

## Introduction

Greater amberjack (*Seriola dumerili*) is a cosmopolitan seawater fish species, and its farming and consumption have dramatically increased in recent years (1), which has attracted significant attention due to its repute as a good source of protein, EPA and DHA, and high acceptance in the worldwide market (2). However, greater amberjack, like other seafood, is prone to spoilage during storage. Microorganism growth is an important factor in seafood spoilage (3, 4). Therefore, there is a need to develop methods for suppressing the growth of microorganisms in seafood to maintain its quality.

Modified atmosphere packaging (MAP) is an effective strategy for food preservation that inhibits microbial growth, decreases lipid oxidation, and protein degradation (5, 6). Previous studies have shown that MAP improves the quality of fish and fishery products by inhibiting bacterial growth (7, 8). And some researchers have revealed that the use of a mixture of N_2_ and CO_2_ in food packaging more effectively extends the food shelf life compared with O_2_ (5, 8–10). The aerobic plate count of Golden pompano did not exceed the acceptable levels after 30 days of storage by treatment with MAP (70% CO_2_/30% N_2_) during superchilling storage, whereas the microbiologically exceeded acceptable levels on day 26 in the air packing groups (6). Similarly, the growth of aerobic plate count and psychrotrophic bacterial counts in Atlantic salmon filets in MAP (60% CO_2_/40% N_2_) treatment groups was slower than in air packing groups during chilled storage, which could effectively maintain the quality of Atlantic salmon filets (11). ε-polylysine (PL) is a natural food preservation additive, produced through the fermentation of *Streptomyces albulus* (12). PL has strong antimicrobial activity against Gram-positive and Gram-negative bacteria, such as *Escherichia coli*, *Salmonella* sp., and *Staphylococcus aureus* (13, 14), which are generally recognized as safe (GRAS) by the FDA in the U.S. (GRN No. 336) (15). The ability of PL to suppress bacterial growth and extend the shelf life of seafood and seafood products was confirmed in previous studies (3, 16, 17). However, there has been no research on the effect of PL, MAP, and their combinations on the quality and microbial community of farmed greater amberjack filets during chilled storage.

To address this deficiency, the effects of PL, MAP, and their combination on the physicochemical indicators of farmed greater amberjack filets during storage at 4°C were evaluated in this study. Changes in the microbial community were investigated by high-throughput 16S rRNA gene sequencing. Understanding the effects of PL, MAP, and combination treatment on the changes in microbiological and physicochemical properties is aimed at providing an effective preservation strategy for extending the shelf life of farmed greater amberjack filets.

## Materials and methods

### Sample preparation and treatment

Farmed greater amberjack (weighing 2200 ± 120 g) was purchased from a local aquatic market in Guangzhou, China, and transported to our laboratory on ice in a large tank within 30 min. The greater amberjack was slaughtered using head shot, scaled, gutted, deskinned, and sliced into filets on a clean bench (weight of 200 ± 10 g). Moreover, all the processing steps were carried out with sterilized tools, and all workers wore sterilized masks and gloves. The filets were then washed in sterilized water, drained through sterilized stainless steel wire mesh for 5 min, and randomly separated into four groups. The four groups were performed as follows: (1) control; filets were soaked in sterilized water for 15 min, drained on a clean bench for 10 min on sterilized stainless steel wire mesh, and packaged with atmospheric air. (2) PL; filets were soaked in 0.5% (w/v) PL for 15 min, drained, and packaged with atmospheric air. (3) MAP; filets were soaked in sterilized water for 15 min, drained, and packaged with mixed gas. (4) MAP + PL; filets were soaked in 0.5% (w/v) PL for 15 min, drained, and packaged with mixed gas. Sterilized soaking water and PL solutions were prepared at 4°C, and the filet/soaked solution ratio was 1:5 (w/v). Food grade PL (purity ≥ 99%) was purchased from Bainafo Biotechnology Co., Ltd. (Zhengzhou, China). The bags in the control and PL groups were sealed and packaged with atmospheric air. Air was first removed from the MAP and MAP + PL groups, then the 40% CO_2_ and 60% N_2_ gas mixture were injected, and the bags were sealed using a packaging machine (MAP-D400; Suzhou Senrui Co., Ltd., Suzhou, China). The initial gas and filet ratio were approximately 4:1 (v/w) for the atmospheric air and MAP treatment groups to ensure a relatively stable composition of gas during greater amberjack storage. Specifically, control and PL groups were packaged in a normal polypropylene bag, while MAP and MAP + PL groups were packaged in high gas barrier performance bags with a low oxygen transmission rate of 0.42 cm^3^ m^–2^ day^–1^ atm^–1^ at 22°C (Sinopharm Chemical Reagent Co., Ltd., Shanghai, China). For each treatment group, a total of 120 individually packed filets were prepared and stored at 4°C for 12 days. Sampling bags were randomly selected from each group at each sampling time.

### Microbial enumeration

The microbial analysis was performed using the method described by Li et al. (18). At each sampling time, three greater amberjack filet packets from each group were selected randomly for the analysis of microbial changes. A 25 g sample of greater amberjack filets was aseptically weighed and homogenized with 225 mL of sterile 0.85% normal saline in a stomacher bag for 3 min using a paddle blender (Stomacher^®^ 3500, Thermo Bioreactor, Seward Ltd., Worthing, UK). The homogenized solutions were serially diluted with 0.01 M PBS (pH 7.2) and 100 μL of the resultant serial solution was spread onto Tryptic soy agar plates (TSA, Huankai Microbial Sci. & Tech. Co., Ltd., Guangdong, China). The plates were incubated at 25°C for 48 h for total viable counts (TVC), and at 7°C for 10 days for psychrotrophic bacterial counts, respectively.

### Determination of pH and total volatile basic nitrogen

The greater amberjack samples (10 g) were minced using a hand blender (JBQ-B50M2; Bear, Guangdong, China). Five grams of minced meat was homogenized with 50 mL of deionized water for 30 s using a vortex mixer (VOETEX2, IKA, Germany) and shaken on a gyratory shaker at 100 rpm for 30 min at room temperature. The homogenized mixture was centrifuged at 2,800 × *g* for 5 min at 4°C. The pH of the supernatant was measured using a digital pH meter (FiveEasy Plus™; Mettler Toledo, China). Moreover, 5 mL of the supernatant was equally mixed with 10 g/L of magnesium oxide in a Kjeldahl tube, and total volatile basic nitrogen (TVB-N) was measured using the Kjeldahl nitrogen apparatus (Kjeldahl™ 2300, Foss, Denmark), as previously described (19). The results were expressed in units of mg N/100 g.

### Sensory evaluation

The sensory analysis of the fish filet was performed according to a previous study (20). Ten trained panelists (five women and five men between 22 and 35 years old) in our Sensory Evaluation Center were trained according to the Codex Alimentarius (21). The texture, odor, and color of raw filets were evaluated according to China’s Guidelines For The Sensory Evaluation Of Aquatic Products (GB/T 37062-2018) (22) using a 5-point descriptive scale: odor, 5 = fresh, 4 = slight fresh, 3 = moderate, 2 = slight off-odor, and 1 = off-odor; color, 5 = bright, 4 = slight bright, 3 = moderate, 2 = slight dull, and 1 = dull; texture, 5 = tight and resilient, 4 = slight loose and slight resilient, 3 = moderate, 2 = loose and soft, and 1 = very loose and very soft. The sensory index (SI) was calculated according to the previous study (18, 23), SI = (2 × odor + 2 × color + texture)/5. The greater amberjack samples were considered sensory rejection points whenever the score was found to be less than 2.5 (18, 23, 24). The sensory evaluation was approved by the ethics committee of the Sensory Evaluation Center, Key Laboratory of Aquatic Product Processing, Ministry of Agriculture and Rural Affairs of China, South China Sea Fisheries Research Institute (approval date 10 April 2022).

### Determination of biogenic amines

Five grams of greater amberjack minced meat was mixed with 20 mL of 0.4 M cold perchloric acid, homogenized, and centrifuged at 10,000 × *g* for 10 min. The supernatant (1 mL) was mixed with 100 μL of 2 M sodium hydroxide and 300 μL of saturated sodium bicarbonate, and 1 mL of dansyl chloride was added (10 mg/mL), followed by incubation at 40°C for 45 min in the dark for derivatization. After the derivative reaction, biogenic amines were analyzed using HPLC. The 20 μL of samples solutions were injected into HPLC (LC-20AD; Shimadzu, Japan) equipped with a reversed-phase chromatographic column (ChromCore™ C18, 250 mm × 4.6 mm; Nano Chrome, China) and an SPD-M20A ultraviolet detector (Shimadzu, Japan) at 254 nm wavelength. The 0.1 M ammonium acetate and 100% acetonitrile were used as gradient elution solvent A and solvent B. Gradient elution program was performed as previously described (25).

### Texture measurement

The texture of the greater amberjack filets was analyzed using a texture analyzer (T-25; IKA, Staufen, Germany) and a TA44 probe (4-mm diameter) was used to measure the fish sample pieces (2 cm × 2 cm × 1 cm). Hardness, chewiness, and springiness were measured by texture profile analysis (TPA) mode with test speed of 1 mm/s, test distance of 5 mm, and trigger force of 5.0 g (24). Each sample was tested six times.

### Microbial community analysis

Microbial community analysis was performed as described previously (26). Ten grams of greater amberjack flesh were homogenized with 20 mL of 0.85% sterile saline at different storage times. The homogenized solutions were centrifuged at 150 × *g* for 7 min to remove fish particles, and the supernatant was obtained and centrifuged at 12,000 × *g* for 10 min at 4°C. The resulting pellet was used for DNA extraction. DNA was extracted using the Magnetic Soil and Stool DNA Kit (TIANGEN BIOTECH Co., LTD., Beijing, China), according to the manufacturer’s protocol. DNA quality was checked using 1% agarose gel electrophoresis. The 27F (5′-AGAGTTTGATCCTGGCTCAG-3′) and 1492R (5′-ACCTTGTTACGACTT-3′) primers were used to amplify the 16S rRNA gene using Kapa HiFi HotStart ReadyMix (Kapa Biosystems, Wilmington, MA, USA). Each 20 μL of PCR mixture contained 10 ng template DNA, 2 μL of dNTPs (2.5 mM), 0.4 μL of DNA polymerase (5 U/μL), 4 μL of 5 × PCR buffer, 0.8 μL of forward primer (5 μM), and 0.8 μL of reverse primer (5 μM). The PCR amplification conditions were as follows: 95°C for 2 min, 30 cycles of 95°C for 30 s, 55°C for 30 s, 72°C for 1 min 30 s, and a final extension for 7 min at 72°C. The PCR amplicons were purified using Agencourt AMPure XP Beads (Beckman Coulter, Indianapolis, IN, USA) and quantified using a Qubit 4.0 fluorometer (Invitrogen, Thermo Fisher Scientific, OR, USA). Subsequently, the SMRT bell libraries were constructed from PCR amplicons using the SMRT bell Template Prep Kit 2.0, according to the manufacturer’s instructions (Pacific Biosciences). Purified SMRTbell libraries from the pooled and barcoded samples were sequenced using the Sequel II Sequencing kit 2.0 on a single PacBio Sequel II 8M cell. The raw reads were filtered and demultiplexed using SMRT Link software (version 8.0) (PacBio) to obtain circular consensus sequencing (CCS) reads. CCS reads were sorted into different samples based on barcodes. After removing the primer and barcodes, high-quality sequences were extracted using the Quantitative Insights into Microbial Ecology (QIIME) package (version 1.7) (27). PyNAST (28) and UCLUST (29) were used to align and identify high-quality sequences based on previous studies (30). Subsequently, a threshold of 97% identity was classified into the same operational taxonomic unit (OTU) using UCLUST (29). Potential chimeric sequences in the representative set of OTUs were removed using the ChimeraSlayer software.^1^ Taxonomy of the OTUs was classified using Greengenes databases (version 13.8) (31), Ribosomal Database Project (32), and SILVA databases (33) with a minimum bootstrap threshold of 80%.

### Statistical analysis

All measurements were performed at least in triplicate and are presented as mean ± standard deviation. The data were analyzed using one-way ANOVA with multiple comparisons of Tukey HSD tests. Statistical significance was set at *P* < 0.05.

## Results and discussion

### Microbial enumeration

Microorganisms play a key role in fish spoilage. Changes in TVC and psychrophilic bacteria observed in the different treatment groups during refrigerated storage are shown in Table 1. The initial TVC of the control was 4.30 ± 0.02 log CFU/g. After treatment with PL, the TVC reduced to 4.15 ± 0.11 log CFU/g in PL groups, and significantly decreased to 4.11 ± 0.06 log CFU/g in MAP + PL groups at 0 days, respectively. Similar to our results, the total aerobic counts decreased by 1.0 log CFU/g after treatment with PL in bighead carp filets at 0 days of storage at 4°C (34). According to 7 log CFU/g as the permissible level of TVC in fisheries (35), the control, PL, MAP, and MAP + PL treatment groups exceeded the permissible level after storage for 6, 10, 12, and 12 days, respectively. Treatment with PL, MAP, and MAP + PL prolonged the microbiological shelf-life of greater amberjack fillets by 4, 6, and 6 days compared with control groups, respectively. Previous studies also revealed that treatment with 0.1% PL could extend the shelf life of Pacific white shrimp through suppressing the growth rate of TVC (17). A gas ratio of 70% CO_2_/30% N_2_ extended the microbiological shelf life of golden pompano filets by 5 days (6). As shown in Table 1, the changes in psychrotrophic bacterial counts were similar to those in the TVC. An increase in the number of storage days caused a steady increase in psychrotrophic bacterial counts in all the groups. After 12 days of storage, the psychrotrophic bacterial counts reached 8.41 ± 0.36, 7.42 ± 0.36, 7.02 ± 0.19, and 6.45 ± 0.20 log CFU/g in control, PL, MAP, and MAP + PL groups, respectively. Compared with the control groups, PL and MAP extended shelf life by suppressing microbial growth in greater amberjack filets. Previous studies have also reported that PL treatment controlled microorganism growth to extend the shelf life of seafood, such as bighead carp (*Aristichthys nobilis*) (34), Pacific white shrimp (*Litopenaeus vannamei*) (17), and large yellow croaker (*Pseudosciaena crocea*) (3). In addition, MAP with N_2_ and CO_2_ had an extended fish shelf life ability, which was attributed to its high ability to suppress microorganism growth (36–38). All treatment groups were effective in reducing the TVC in the samples throughout the cold storage when compared to the control. Among the treatment groups, MAP + PL was shown to be the most effective treatment in delaying microbial growth. The superiority of the MAP + PL treatment over the PL treatment was shown by significantly lowering TVC after 6 days of storage and over the MAP treatment after 8 days of storage. Therefore, the combination of MAP and PL is more effective than individual treatment in controlling the microbial growth in the fish samples.

**TABLE 1:** Changes in total viable counts and psychrotrophic bacterial counts of greater amberjack during storage at 4°C.

	Treatments	Storage days (days)						
		0	2	4	6	8	10	12
Total viable counts (TVC) (log CFU/g)	Control	4.30 ± 0.02^Af^	5.18 ± 0.02^Ae^	6.34 ± 0.14^Ad^	7.24 ± 0.10^Ac^	7.32 ± 0.23^Ab^	7.91 ± 0.20^Ab^	8.84 ± 0.03^Aa^
	PL	4.15 ± 0.11^ABf^	4.18 ± 0.03^Bf^	4.75 ± 0.06^Be^	5.88 ± 0.04^Bd^	6.52 ± 0.40^Bb^	7.15 ± 0.09^Ab^	7.94 ± 0.26^Ba^
	MAP	4.27 ± 0.03^ABf^	4.25 ± 0.05^Bf^	4.54 ± 0.12^Be^	4.82 ± 0.04^Cd^	5.64 ± 0.09^Cb^	6.74 ± 0.09^Bb^	7.27 ± 0.16^Ca^
	MAP + PL	4.11 ± 0.06^Be^	4.08 ± 0.13^Be^	4.54 ± 0.08^Bd^	4.66 ± 0.04^Ccd^	4.96 ± 0.08^Db^	6.21 ± 0.18^Cb^	7.06 ± 0.11^Ca^
Psychrotrophic bacterial counts (log CFU/g)	Control	3.53 ± 0.04^Ae^	3.55 ± 0.08^Ae^	5.63 ± 0.02^Ad^	6.79 ± 0.15^Ac^	7.00 ± 0.12^Ac^	7.89 ± 0.24^Ab^	8.41 ± 0.36^Aa^
	PL	2.79 ± 0.42^ABe^	3.16 ± 0.30^ADe^	3.89 ± 0.15^Bd^	4.88 ± 0.08^Bc^	6.38 ± 0.11^Bb^	6.90 ± 0.23^Bab^	7.42 ± 0.36^Ba^
	MAP	3.32 ± 0.10^ABe^	3.53 ± 0.09^Ae^	4.20 ± 0.05^Cd^	4.46 ± 0.11^Cd^	4.92 ± 0.06^Cc^	6.45 ± 0.29^Bb^	7.02 ± 0.19^BCa^
	MAP + PL	2.57 ± 0.42^Bc^	2.68 ± 0.17^Bc^	3.69 ± 0.16^Cb^	4.23 ± 0.14^Cb^	4.28 ± 0.22^Dd^	6.28 ± 0.31^Ba^	6.45 ± 0.20^Ca^

ND, not detection; control, packed under atmospheric air; PL, ε-polylysine; MAP, modified atmosphere packaging; PL + MAP, combination of ε-polylysine and modified atmosphere packaging. Different uppercase letters indicate significant differences between treatments, and different lowercase letters indicate significant differences within treatments (*P* < 0.05).

### Analysis of pH, sensory index, and total volatile basic nitrogen

**FIGURE 1:**
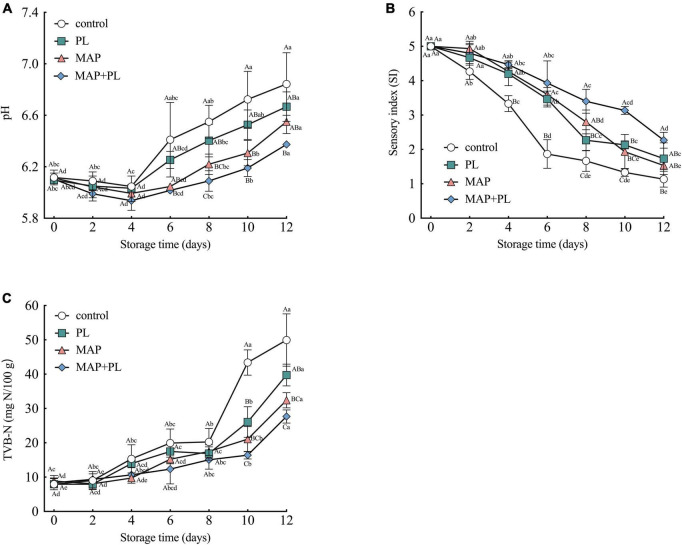
Changes in pH **(A)**, TVB-N **(B)**, and sensory index (SI) **(C)** of greater amberjack during storage at 4°C. Control, packed under atmospheric air; PL, ε-polylysine; MAP, modified atmosphere packaging; MAP + PL, combination of modified atmosphere packaging and ε-polylysine. Different uppercase letters indicate significant differences between treatments, and different lowercase letters indicate significant differences within treatments (*P* < 0.05).

The changes in physicochemical properties and sensory parameters, including pH, TVB-N, and SI for the control, PL, MAP, and MAP + PL groups are shown in Figure 1. All groups had similar pH profiles, with pH values gradually decreasing from 0 to 4 days in the control and PL groups, and from 0 to 6 days in the MAP and MAP + PL groups. With an extension of the storage period, the pH values recovered in all groups, finally reaching 6.84 ± 0.24, 6.67 ± 0.12, 6.55 ± 0.09, and 6.37 ± 0.02 in the control, PL, MAP, and MAP + PL groups, respectively (Figure 1A). The decreasing trend in pH at the beginning of storage could have been due to lactic acid production and glycolysis, and the accumulation of biogenic amines and TVB-N, leading to pH recovery during the extension of the storage period (18). The final pH values in the treatment groups were lower than those in the control groups at the end of storage, and the MAP + PL treatment groups were significantly lower than those in the control groups, which indicated that the spoilage progress of the treatment groups was slower than that of the control group.

The results of the sensory analysis are shown in Figure 1B and Supplementary Table 1. The SI was the highest for all samples on the first day of storage. The SI score decreased as the storage time progressed, indicating that the greater amberjack underwent spoilage during storage. The SI scores of the control decreased from 5.00 to 1.86 after 6 days of storage, which was lower than the acceptable level of 2.5. Moreover, the greater amberjack filets from the PL, MAP, and MAP + PL groups became unacceptable at storage time points of 8, 10, and 12 days with values of 2.27, 1.93, and 2.26, respectively, and the SI values of the MAP + PL groups were significantly different from the control groups after 12 days of storage. The general higher SI scores throughout the storage indicated that all treatments were effective in retaining the sensory quality of the filets when compared to the control, in which the MAP + PL was the most effective in retaining the fish quality throughout the 12 days of storage.

Total volatile basic nitrogen values are usually associated with the deterioration of fish and fish product quality (23). As shown in Figure 1C, the TVB-N of the fresh greater amberjack was approximately 8 mg N/100 g, which increased in all samples with increasing storage duration. After 10 days of storage, the TVB-N value reached 43.40 ± 3.67 mg N/100 g in the control group. Various studies have reported that the permissible level of TVB-N is 30 mg N/100 g (20, 39, 40). While the TVB-N values of PL and MAP groups were 26.07 ± 4.46 mg N/100 g and 21.07 ± 3.65 mg N/100 g after 10 days of storage, they increased to 39.73 ± 3.18 mg N/100 g and 32.40 ± 2.23 mg N/100 after 12 days of storage, respectively. Furthermore, the TVB-N value of the MAP + PL group was 27.67 ± 1.92 mg N/100 g after 12 days of storage. The differences in the TVB-N values between the control and treatments were evident after prolonged storage. All treatments yielded filets with lower TVB-N than the control after 10 days of storage. Among the treatments, the filets treated with a combination of MAP + PL yielded significantly lower TVB-N values than the PL group, and also lower, although not statistically significant, than the MAP group. This phenomenon could be attributed to a lack of oxygen, which restricts the growth of aerobic spoilage bacteria under CO_2_ conditions, leading to low TVB-N values. The TVB-N value in the MAP + PL groups did not exceed the acceptable levels after 12 days of storage, but the SI was lower than the set acceptable level of 2.5. This result corresponds with the previous studies, which also reported that TVB-N was low despite sensory analysis results indicating fish spoilage (7, 41). We hypothesize that the increase in TVB-N values is caused by the activity of some spoilage bacteria capable of producing unpleasant odors, discoloring, or texture deterioration without increasing TVB-N values (41). Therefore, the spoilage of fish filets requires comprehensive evaluation based on multiple properties.

### Biogenic amines

The accumulation of biogenic amines in seafood results from microorganisms decarboxylating free amino acids into biogenic amines (25, 42). Some biogenic amines such as cadaverine (CAD) and putrescine (PUT) have a strong unpleasant odor and are often associated with the putrefaction of animal tissue. There are no legal limits for these amines in food, probably due to their offensive odor, limiting the risk of consumers’ exposure to the harmful levels of these amines in food (43). Changes in biogenic amine concentrations (mg/kg) of the control and treated groups in the greater amberjack filets during storage at 4°C are shown in Table 2. The concentration of CAD significantly increased in the control and PL groups after 4 days of storage, reaching 48.29 ± 0.63 mg/kg and 26.75 ± 0.94 mg/kg, respectively. At end of storage, the concentration of CAD was increased to 169.52 ± 14.90 mg/kg and 172.18 ± 6.91 mg/kg in control and PL groups. In contrast, the increase in the CAD levels was delayed in the MAP and MAP + PL groups, in which the CAD concentrations were significantly increased after 6 days of storage, finally reaching 106.83 ± 8.16 mg/kg and 86.71 ± 9.73 mg/kg after 12 days of storage in MAP and MAP + PL groups, respectively. The lowest concentration of CAD was observed in the MAP + PL groups at the end of storage. The PUT concentrations in the treatment groups were lower than those in the control groups at the end of storage. The lowest concentration was observed in the MAP + PL treatment groups, which was significantly lower than the control and other treatment groups. Histamine (HIS) was not detected at the beginning of storage, and it significantly increased to 109.10 ± 2.18 mg/kg at 12 days in the control groups. PL and MAP treatments significantly suppressed the histamine accumulation, which finally increased to 66.21 ± 4.99 mg/kg, 40.10 ± 4.20 mg/kg, and 39.48 ± 1.76 mg/kg in PL, MAP, and MAP + PL groups at the end of storage. For USFDA, a dose of 50 mg/kg of HIS was set as an indicator for decomposition, and 200 mg/kg was the hazard level (44). According to USFDA, the HIS concentration in all the groups is within the safe levels, but above the 50 mg/kg quality standard for control and PL treatment groups at the end of storage. Based on the biogenic amines analysis results, the PL, MAP, and MAP + PL treatments could delay the accumulation of biogenic amines compared with the control groups, which correlated well with the fact that treatment with PL, MAP, and MAP + PL could delay microorganism growth.

**TABLE 2:** Changes in biogenic amines concentration (mg/kg) of control and treated groups in greater amberjack filets during storage at 4°C.

Biogenic amines (mg/kg)	Treatments	Storage days (days)						
		0	2	4	6	8	10	12
Cadaverine (CAD)	Control	0.38 ± 0.08^Ae^	2.23 ± 0.14^Ae^	48.29 ± 0.63^Ad^	65.59 ± 1.53^Ad^	110.91 ± 5.67^Ac^	130.44 ± 6.10^Ab^	169.52 ± 14.90^Aa^
	PL	0.43 ± 0.05^Ae^	1.77 ± 0.23^ABe^	26.75 ± 0.94^Bd^	36.06 ± 3.57^Bd^	75.16 ± 2.52^Bc^	127.46 ± 9.51^Ab^	172.18 ± 6.91^Aa^
	MAP	0.38 ± 0.00^Ae^	1.33 ± 0.21^Be^	6.81 ± 0.72^Cde^	17.52 ± 1.26^Cd^	45.28 ± 2.94^Cc^	89.88 ± 7.57^Bb^	106.83 ± 8.16^Ba^
	MAP + PL	0.36 ± 0.01^Ae^	0.65 ± 0.13^Ce^	1.71 ± 0.19^Dde^	16.55 ± 2.90^Ccd^	31.31 ± 6.64^Dc^	58.11 ± 8.38^Cb^	87.01 ± 9.73^Ba^
Putrescine (PUT)	Control	0.76 ± 0.08^Ae^	1.32 ± 0.29^Ae^	4.61 ± 0.71^Ae^	64.81 ± 10.57^Ad^	95.11 ± 7.11^Ac^	130.80 ± 5.48^Ab^	183.65 ± 10.33^Aa^
	PL	0.74 ± 0.06^Ae^	1.30 ± 0.26^Ae^	4.31 ± 0.23^Ae^	34.36 ± 4.22^Bd^	63.21 ± 3.37^Bc^	88.40 ± 0.79^Bb^	121.67 ± 5.47^Ba^
	MAP	0.73 ± 0.08^Ae^	0.86 ± 0.10^ABe^	1.18 ± 0.32^Be^	3.50 ± 0.32^Ce^	31.40 ± 2.23^Cc^	73.31 ± 2.70^Cb^	110.30 ± 17.29^BCa^
	MAP + PL	0.68 ± 0.28^Ad^	0.56 ± 0.15^Bd^	1.06 ± 0.06^Bd^	3.39 ± 0.42^Cd^	27.88 ± 5.24^Cc^	61.30 ± 2.83^Db^	83.29 ± 1.75^Ca^
Histamine (HIS)	Control	ND	0.03 ± 0.02^Ad^	0.18 ± 0.05^Ad^	31.21 ± 11.63^Ac^	84.53 ± 9.40^Ab^	97.51 ± 5.70^Aab^	109.10 ± 2.18^Aa^
	PL	ND	0.03 ± 0.02^Ae^	0.04 ± 0.02^Be^	10.17 ± 1.92^Bd^	23.54 ± 2.90^Bc^	58.19 ± 2.44^Bb^	66.21 ± 4.99^Ba^
	MAP	ND	0.07 ± 0.03^Ad^	0.06 ± 0.01^Bd^	5.53 ± 0.78^Bcd^	10.90 ± 1.74^BCc^	28.38 ± 2.59^Cb^	40.10 ± 4.20^Ca^
	MAP + PL	ND	0.04 ± 0.02^Ad^	0.07 ± 0.00^Bd^	2.93 ± 0.57^Bcd^	4.61 ± 1.15^Cc^	19.43 ± 2.02^Cb^	39.48 ± 1.76^Ca^

ND, not detection; control, packed under atmospheric air; PL, ε-polylysine; MAP, modified atmosphere packaging; PL + MAP, combination of ε-polylysine and modified atmosphere packaging. Different uppercase letters indicate significant differences between treatments, and different lowercase letters indicate significant differences within treatments (*P* < 0.05).

### Texture

**FIGURE 2:**
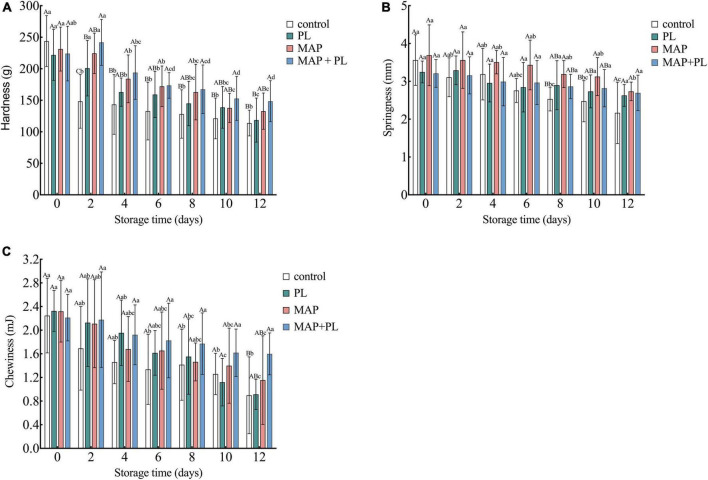
Changes in texture properties including hardness **(A)**, springiness **(B)**, and chewiness **(C)** of greater amberjack during storage at 4°C. Control, packed under atmospheric air; PL, ε-polylysine; MAP, modified atmosphere packaging; MAP + PL, combination of modified atmosphere packaging and ε-polylysine. Different uppercase letters indicate significant differences between treatments, and different lowercase letters indicate significant differences within treatments (*P* < 0.05).

Similar to SI analysis, texture analysis has also been used to evaluate the quality of fish filets in previous studies (23, 24, 45). The hardness, chewiness, and springiness of the greater amberjack filets during storage are shown in Figure 2. Hardness is the most important texture index of fish filets (24, 45). The hardness of the greater amberjack decreased with increasing storage time in all groups. After 12 days of storage, the hardness of greater amberjack decreased to 113.18, 118.64, 132.75, and 148.53 g in control, PL, MAP, and MAP + PL groups, respectively (Figure 2A). The increasing gaps between muscle fibers and loss of myofibers lead to a decrease in the hardness value (27). Similar to hardness, the chewiness and springiness of the greater amberjack were also shown to decline trends in all groups (Figures 2B,C). The decreased speed in the PL, MAP, and MAP + PL groups was slower than that in the control groups, especially the combination of MAP and PL, which could be attributed to the slower microbial growth rate. Previous studies revealed that microorganism growth causes the texture indices to decline in seafood (34, 37). Moreover, the gas composition in the MAP treatment did not contain O_2_ in this study, which might have reduced the protein oxidation of fish filets to reduce the deterioration of texture properties during storage (6, 7).

### Microbial community

**FIGURE 3:**
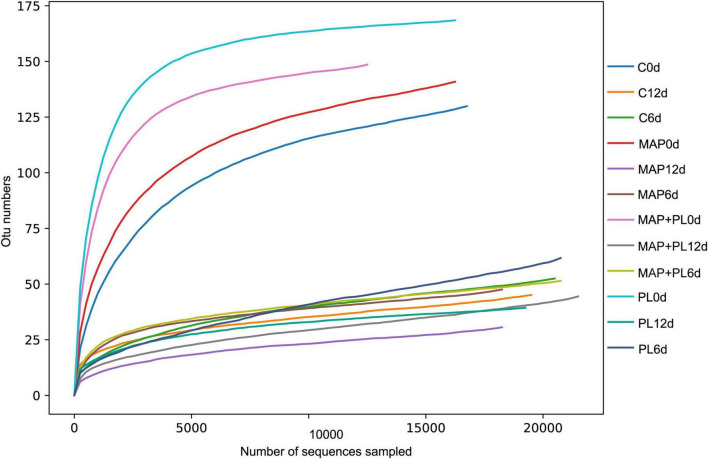
Rarefaction curve of microbiota in greater amberjack filets during storage at 4°C. Control, packed under atmospheric air; PL, ε-polylysine; MAP, modified atmosphere packaging; MAP + PL, combination of modified atmosphere packaging and ε-polylysine.

**FIGURE 4:**
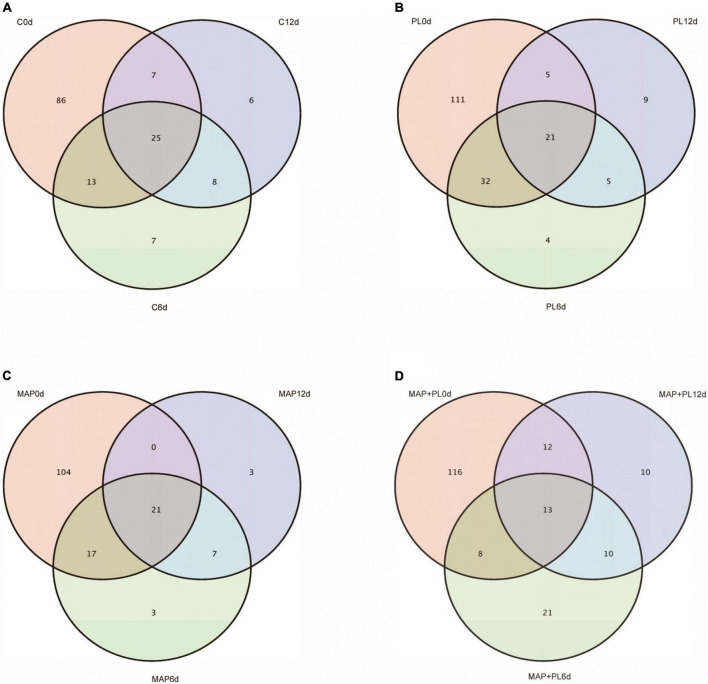
Venn diagram showing the distribution of the OTUs in control **(A)**, PL **(B)**, MAP **(C)**, and MAP + PL **(D)** of greater amberjack during storage at 4°C. Control, packed under atmospheric air; PL, ε-polylysine; MAP, modified atmosphere packaging; MAP + PL, combination of modified atmosphere packaging and ε-polylysine.

**FIGURE 5:**
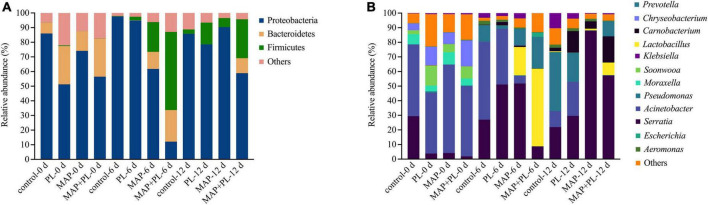
Microbial community succession at phylum **(A)** and genus **(B)** levels of greater amberjack during storage at 4°C. Control, packed under atmospheric air; PL, ε-polylysine; MAP, modified atmosphere packaging; MAP + PL, combination of modified atmosphere packaging and ε-polylysine.

16S rRNA gene sequencing data were clustered into OTUs. The rarefaction curves tended to reach the saturation plateau, indicating that the data size of the sequences was reasonable (Figure 3). To visually describe the OTUs under different treatments, the OTUs in the control, PL, MAP, and MAP + PL groups of the greater amberjack samples were plotted on a Venn diagram (Figure 4). The Venn diagram shows a marked decrease in the number of OTUs after 6 and 12 days of storage in all groups. The changes in microbial taxonomic composition at the phylum and genus levels of the greater amberjack during storage are shown in Figure 5. Proteobacteria was the main phylum observed during the storage in all groups (Figure 5A). A total of 12 microbial genera, with a relative abundance of over 1%, were observed in the storage stages (Figure 5B). The composition of the initial microbiota was more diverse than that observed at later storage points. With increasing storage time, the abundance of *Chryseobacterium*, *Soonwooa*, and *Moraxella* decreased. *Acinetobacter* is the most prevalent genus in the flesh filet before storage. In the control group, *Acinetobacter* (53.42%) has become the dominant bacterial at 6 days of storage. *Pseudomonas* was relatively rare in the control group before storage but gradually increased with storage time to 40.3% becoming the predominant species in the control group at the end of storage at 12 days. *Pseudomonas* (19.95%), *Serratia* (29.60%), and *Acinetobacter* (23.36%) became dominant in the PL treatment group at the end of storage. *Acinetobacter* is an aerobic species, which was usually found in flesh fish or in the middle or end of storage but not as the dominant spoilage bacterial (46). Indeed, due to *Acinetobacter* not having the ability to produce high levels of biogenic amines and cannot hydrolyze proteins, this species did not belong to the stronger spoilage bacterium (47–50). Therefore, *Pseudomonas* might be the main contributor to the spoilage of fish filets in control groups, and treatment with PL might be extending the shelf life of fish filets by suppressing the growth of *Pseudomonas*. Similar results were found that treatment with PL could extend the shelf life of Pacific white shrimp by inhibiting the growth of *Pseudomonas* (17). Furthermore, the *Pseudomonas* species have been reported to be dominant spoilage microorganisms in food (17). *Pseudomonas* was 0.15 and 0.07% of the total bacteria after 12 days of storage in the MAP and MAP + PL treatment groups, respectively, demonstrating that the MAP treatment could extend the shelf life of greater amberjack filets by restricting the growth of *Pseudomonas*. In the MAP and MAP + PL treatment groups, *Serratia* was 4.23 and 1.90% of the total bacteria at the beginning of the storage period, respectively. With increasing storage time, *Serratia* increased to 51.81 and 8.72% in the MAP and MAP + PL groups, respectively, after 6 days of storage. *Serratia* was the predominant species identified in greater amberjack with 87.9 and 57.3% of the total genus at the end of storage in the MAP and MAP + PL treatment groups, respectively, which indicated that *Serratia* played a vital role in the spoilage process in MAP and MAP + PL groups. Various *Serratia* strains were able to grow under CO_2_ and N_2_ conditions (51). Previous studies have also reported that *Serratia* was an important bacterium associated with seafood spoilage (52, 53). Interestingly, the *Carnobacterium* was 14.73 and 17.99% in PL and MAP + PL groups at the end of storage. We hypothesized that since the predominant spoilage bacterium was restricted, the *Carnobacterium* might play a role in filets and shown in some points of storage under PL treatments, but the reason is not clear yet and needs further deep study.

## Conclusion

In this study, the effects of PL, MAP, and MAP + PL on the microbial quality and physicochemical properties of greater amberjack filets during storage were evaluated. All treatments were effective in extending the shelf life of the greater amberjack filets when compared to the control. When SI was compared to TVC, TVB-N, and biogenic amines, the shortest storage duration was obtained. Based on the SI, the shelf life of the filets in the control, PL, MAP, and MAP + PL groups was 4, 6, 8, and 10 days, respectively, indicating the efficiency of the PL, and MAP treatments, and that the combination of the MAP and PL treatment was the most effective preservation strategy in extending the shelf life of the fish filets. High-throughput 16S rRNA gene sequencing showed that the microbial composition and diversity were affected by the PL, MAP, and MAP + PL treatments. The relative abundance of *Pseudomonas* and *Serratia* were more inhibited under treatment with MAP + PL treatment compared with other groups. Our study suggests that the MAP + PL treatment could serve as an effective preservation strategy to extend the shelf life of greater amberjack.

## Data availability statement

The datasets presented in this study can be found in online repositories. The names of the repository/repositories and accession number(s) can be found below: https://www.ncbi.nlm.nih.gov/bioproject/PRJNA887851, PRJNA887851.

## Author contributions

DW: data curation, methodology, validation, writing – original draft, visualization, and funding acquisition. XL: data curation, methodology, and formal analysis. XY: project administration and supervision. SC and YW: methodology and software. LL: formal analysis and writing – review and editing. CP: formal analysis and resources. YZ: writing – review and editing and funding acquisition. All authors contributed to the article and approved the submitted version.

## Footnotes

1
https://packages.debian.org/bullseye/chimeraslayer
