# Supplementary material for: Unraveling the effect of the combination of modified atmosphere packaging and ε-polylysine on the physicochemical properties and bacterial community of greater amberjack (Seriola dumerili)
Source: Front Nutr. 2022 Nov 18;9:1035714. doi: 10.3389/fnut.2022.1035714 (PMC9715602; doi:10.3389/fnut.2022.1035714)
Supplement: Supplementary file 1 [file Table_1.docx]

Table S1 Changes in texture properties including odor, color, and texture of greater amberjack during storage at 4 °C.

|  | Treatments | Storage days (days) | | | | | | |
| --- | --- | --- | --- | --- | --- | --- | --- | --- |
|  |  | 0 | 2 | 4 | 6 | 8 | 10 | 12 |
| Odor | control | 5.00±0.00^Aa^ | 4.00±0.00^Aa^ | 2.33±0.50^Ab^ | 1.33±0.50^Abc^ | 1.33±0.50^Abc^ | 1.00±0.00^Ac^ | 1.00±0.00^Ac^ |
|  | PL | 5.00±0.00^Aa^ | 4.67±0.50^Aa^ | 4.00±0.00^Aab^ | 3.00±0.50^Abc^ | 2.67±1.12^Acd^ | 1.67±0.50^Ad^ | 1.67±0.50^Ad^ |
|  | MAP | 5.00±0.00^Aa^ | 5.00±0.00^Ba^ | 4.00±0.00^Aab^ | 3.33±0.87^Abc^ | 3.00±0.50^Abc^ | 2.00±0.87^Acd^ | 1.33±0.50^Ad^ |
|  | MAP+PL | 5.00±0.00^Aa^ | 4.67±0.50^Ca^ | 4.33±0.50^Bab^ | 4.00±0.71^Babc^ | 3.33±0.87^Bbc^ | 3.00±0.71^Bc^ | 1.67±0.87^Ad^ |
| Color | control | 5.00±0.00^Aa^ | 4.67±0.50^Aa^ | 4.00±0.00^Aa^ | 2.00±0.87^Ab^ | 2.00±0.50^Ab^ | 1.67±0.50^Ab^ | 1.33±0.50^Ab^ |
|  | PL | 5.00±0.00^Aa^ | 4.67±0.50^Aa^ | 4.33±0.71^Aa^ | 3.67±0.50^Aab^ | 2.00±0.87^ABbc^ | 2.33±0.50^Ac^ | 1.67±0.71^Bc^ |
|  | MAP | 5.00±0.00^Aa^ | 5.00±0.00^Aa^ | 4.33±0.87^Aa^ | 4.00±0.50^Aab^ | 2.67±0.50^Bbc^ | 2.00±0.87^Ac^ | 1.67±0.87^Bc^ |
|  | MAP+PL | 5.00±0.00^Aa^ | 5.00±0.00^Aa^ | 4.67±0.50^Aab^ | 3.67±1.32^Aab^ | 3.33±0.50^Bab^ | 3.00±0.00^Ab^ | 3.00±1.00^Bb^ |
| Texture | control | 5.00±0.00^Aa^ | 4.00±0.00^Aa^ | 4.00±0.71^Aa^ | 2.67±1.00^Ab^ | 1.67±0.50^Abc^ | 1.33±0.50^Ac^ | 1.00±0.00^Ac^ |
|  | PL | 5.00±0.00^Aa^ | 4.67±0.71^Aa^ | 4.33±1.00^Aa^ | 4.00±0.50^Aab^ | 2.00±0.87^ABbc^ | 2.67±1.00^ABc^ | 2.00±0.71^Ac^ |
|  | MAP | 5.00±0.00^Aa^ | 4.67±0.50^Aab^ | 4.67±0.50^Aab^ | 3.33±1.22^ABbc^ | 2.67±0.87^ABcd^ | 1.67±0.71^Bd^ | 1.67±0.50^ABd^ |
|  | MAP+PL | 5.00±0.00^Aa^ | 4.67±0.50^Aa^ | 4.33±0.71^Aa^ | 4.33±0.50^Ba^ | 3.67±0.50^Ba^ | 3.67±0.50^Ba^ | 2.00±0.50^Bb^ |

Control- packed under atmospheric air; PL- ε-polylysine; MAP- modified atmosphere packaging; PL+MAP- combination of ε-polylysine and modified atmosphere packaging;

Different uppercase letters indicate significant differences between treatments, and different lowercase letters indicate significant differences within treatments (*P* < 0.05).
